# Distinct Longitudinal Contributions of Social Anxiety and Self‐Consciousness to Interpersonal Dysfunction

**DOI:** 10.1002/jclp.70155

**Published:** 2026-05-29

**Authors:** Joseph Maffly‐Kipp, Kaitlyn Nagel, Jay C. Fournier

**Affiliations:** ^1^ Department of Psychiatry The Ohio State University Wexner Medical Center, College of Medicine Columbus Ohio USA; ^2^ Headspace Inc Santa Monica California USA

**Keywords:** internalizing disorders, interpersonal dysfunction, self‐consciousness, social anxiety

## Abstract

We examined whether social anxiety (SA) and self‐consciousness (SC) predicted unique variance in functioning among young adults with internalizing symptoms. We hypothesized that each would independently predict poorer interpersonal functioning and quality of life cross‐sectionally and over 12 months. Participants (*N* = 152; ages: 18–30) included individuals with elevated depression and anxiety symptoms as well as healthy controls. At baseline, participants completed measures of SA, SC, and assessments of quality of life, social/leisure satisfaction, and four domains of interpersonal problems, with reassessments at 6 and 12 months. Baseline SA and SC both uniquely predicted greater interpersonal problems, and SA predicted lower social satisfaction, controlling for depression and anxiety severity. Quality of life was more strongly associated with SC than SA. However, in longitudinal models, all significant effects of baseline SA diminished over time and were no longer significant at the 12‐month follow‐up. In contrast, baseline SC demonstrated more stable relationships with impaired social functioning across the longitudinal period. Our findings suggest that SA may reflect a more state‐like vulnerability that varies with time and circumstances, while SC may be associated with more stable interpersonal problems. These distinctions may ultimately allow clinicians to more effectively separate features associated with shorter‐term interpersonal challenges from those associated with longer‐term problems, and therefore to select more targeted and appropriate treatments.

Social anxiety (SA) and self‐consciousness (SC) both involve maladaptive forms of self‐focused attention. SA is characterized by persistent and intense fears of social judgment, and is common across both clinical and non‐clinical populations (American Psychiatric Association 2013). Contemporary cognitive‐behavioral models emphasize that interpersonal impairment is maintained not only by fear of negative evaluation, but also by maladaptive safety behaviors and post‐event processing that amplify negative self‐impressions and avoidance (Morrison and Heimberg 2013; Spence and Rapee 2016). SC is a key facet of the personality dimension neuroticism that overlaps with SA in several respects, including fears of embarrassment and avoidance of social situations (Costa and McCrae 1992). Trait frameworks conceptualize SC as reflecting chronic self‐evaluative concern and vulnerability to shame and related self‐conscious emotions, which show reliable links with internalizing symptoms (Cândea and Szentagotai‐Tătar 2018). This overlap raises a practical problem: if SA and SC partly reflect a shared self‐evaluative process, then their apparent associations with social functioning may be redundant. Conversely, if they capture meaningfully distinct liabilities (e.g., situational fear of evaluation vs. broader shame‐proneness/self‐evaluative style), separating them could sharpen clinical case conceptualization and risk prediction.

In separate lines of research, both SA and SC have been independently associated with impairments in quality of life and interpersonal functioning, and an increased risk of other internalizing disorders (Buckner et al. 2012; Koyuncu et al. 2014). Moreover, both are associated with a more difficult treatment course and worse functional outcomes in depressed patients (Schneier et al. 2002). Comorbid SA and depression has been associated with greater symptom severity, poorer treatment outcomes, and increased risk for suicidal ideation and attempts (Beesdo et al. 2007; Wersebe et al. 2018). Self‐consciousness has demonstrated a similar relationship with depression, such that SC has been associated with poorer emotion regulation, ineffective use of coping skills, and increased shame (Sullivan et al. 2020; Taylor et al. 2023). Together, individuals with high SA and SC demonstrated worsened symptom severity than SA alone (Hope and Heimberg 1988).

Despite this overlap, it remains unclear whether SC and SA are distinct. They are both characterized by elevated self‐focused attention, fear of evaluation and embarrassment, and behavioral avoidance. However, distinguishing between them could be important and clinically useful. For example, they may present unique challenges to interventional approaches, predict divergent patterns of impairment, or present with distinct comorbidities and symptom profiles. Further, to the extent that they have different temporal trajectories, state‐like symptoms may require different interventions compared to more stable trait‐like characteristics. Separating SC and SA both theoretically and via validated assessments may therefore have direct benefits for improving treatments.

Based on this gap in the literature, we directly compared the predictive power of SC and SA in an adult sample that included both healthy individuals and people with a range of internalizing psychopathology. Although both forms of self‐focused attention have been linked to interpersonal difficulties (Buckner et al. 2012; Koyuncu et al. 2014), few studies have directly compared their relative and unique associations with functional outcomes within the same samples. As a result, it remains unclear whether observed impairments reflect shared variance between the constructs or distinct contributions of each. We specifically tested whether SA and SC each explain incremental variance in quality of life and interpersonal functioning after adjusting for depression and anxiety severity, and whether these associations persist over a 12‐month follow‐up. We hypothesized that SA and SC would each be uniquely associated with lower self‐reported quality of life and impairments in interpersonal functioning both cross‐sectionally and longitudinally over a 12‐month observational window.

## 1 Methods

### 1.1 Participants

Data for the current study were collected at the University of Pittsburgh and The Ohio State University. The initial sample consisted of 152 individuals, ages 18–30 with at least mild symptoms of depression or anxiety (*n* = 96; internalizing group), as measured by the Quick Inventory of Depressive Symptomatology (QIDS; scores greater than or equal to 6) and Patient Reported Outcomes Measurement Information System – Anxiety (PROMIS‐A; scores greater than or equal to 17), as well as individuals with no‐to‐minimal symptoms (*n* = 56; healthy individuals). All procedures were approved by the Institutional Review Board of the relevant institution. All participants provided written informed consent. Sample characteristics can be viewed in Table S1.

### 1.2 Procedure

All participants underwent a clinician‐administered baseline evaluation after eligibility was determined. This evaluation encompassed informed consent, collection of demographic information (including age, educational attainment, racial and ethnic background, and biological sex), and assessment of depressive symptoms, anxiety, and personality characteristics through both self‐report measures and clinician ratings. The baseline assessment was conducted within the framework of a broader investigation into the role of self‐referential processing and emotion regulation in internalizing psychopathology and neuroticism. Additional study components included daily diary assessments and neuroimaging sessions administered during the follow‐up period; however, these elements fall outside the scope of the current investigation and are therefore not discussed further. Participants were also assessed via clinical interview and self‐report questionnaires at 6‐ and 12‐month follow‐ups using a subset of the baseline measures.

### 1.3 Measures

Descriptions and citations for all primary measures can be viewed in Material S1.

#### 1.3.1 Primary Predictor Variables

We used the Liebowitz Social Anxiety Scale (LSAS) to measure SA, and the self‐consciousness subscale of neuroticism from the NEO Personality Inventory—Revised, Self‐Consciousness Subscale to measure SC. For each, higher summed values meant higher levels of SA or SC, respectively.

#### 1.3.2 Primary Outcome Variables

Our primary outcome variables were quality of life, social and leisure satisfaction, and interpersonal functioning. We used the Quality of Life Enjoyment and Satisfaction Questionnaire, the social and leisure subscale of the Social Adjustment Scale Self‐Report, and the Inventory of Interpersonal Problems. The latter has four domains that we examined separately: interpersonal sensitivity, interpersonal ambivalence, need for social approval, and lack of sociability. All primary outcome variables were measured at baseline, 6‐, and 12‐month follow‐up visits.

#### 1.3.3 Covariates

We created depression and anxiety composite scores to account for internalizing symptoms. The depression composite was a z‐scored average of the Montgomery‐Asberg Depression Rating Scale, the Hamilton Depression Rating Scale, the Quick Inventory of Depressive Symptoms, and the depression scale of the Mood and Anxiety Symptom Questionnaire—Short Form (MASQ‐D30), Depression Scale. The anxiety composite was a *z*‐scored average of the anxiety subscale of the MASQ‐D30—Anxiety Scale, the Hamilton Anxiety Rating Scale, and the Patient Reported Outcomes Measurement Information System—Emotional Distress‐Anxiety—Short‐Form.

### 1.4 Analytic Approach

Our primary analyses, conducted using R version 4.4.1 (R Core Team 2024), consisted of multiple regression analyses examining the independent contribution of SA and SC toward the primary outcomes at baseline, controlling for depression, anxiety, age, and sex assigned at birth. We selected multiple regression to test the incremental contribution of SA and SC while accounting for shared variance among predictors.

We next conducted secondary analyses using linear mixed effects models to examine changes in dependent variables across the 12‐month follow‐up interval, focusing longitudinal analyses on estimates at 12 months. All predictors were measured at baseline, and for the longitudinal models we utilized random intercepts to capture the nested structure of the data by participant. We used mixed effects models to account for repeated observations within individuals and to retain all available data under maximum likelihood estimation. By centering the time variable at 12 months, we focus these models on estimates at 12 months, and lower‐order effects of covariates can be interpreted as the relationship between the covariate and the outcome variable 12 months later (Kraemer and Blasey 2004). Interactions with time can be interpreted to suggest that the relationship between the covariate (measured at baseline) and the relevant outcome variable changed across time. In other words, these models tested which effects of SA and SC, if any, remained present after 1 year.

We used False Discovery Rate (FDR) corrections to control for multiple comparisons (*q* = 0.05), with correction applied across all focal social anxiety and self‐consciousness tests within each analysis block. We conducted all analyses first in the full sample (healthy comparison and internalizing groups) and then in the internalizing only sample.

## 2 Results

Bivariate associations between all variables at baseline can be viewed in Table S2 (full sample) and Table S3 (internalizing sample). We observed a moderate correlation between SC an SA in the full sample, *r*(150) = 0.65, *p* < 0.001, although it was lower in the internalizing sample *r*(94) = 0.46, *p* < 0.001.

### 2.1 Cross‐Sectional Associations (Table 1 and Figure 1)

**Table 1:** Results from all cross‐sectional models in the full sample.

	*β*	*b*	*SE*	95% CI	*t*	*p*	*p corrected*
*Social and leisure activities*							
Age	−0.139	−0.026	0.01	[−0.05, −0.01]	−2.60	**0.010**	—
Sex at birth	−0.126	−0.078	0.07	[−0.22, 0.06]	−1.11	0.271	—
Depression	0.387	0.267	0.07	[0.13, 0.40]	3.82	**< 0.001**	—
Anxiety	0.025	0.017	0.06	[−0.11, 0.14]	0.28	0.781	—
Social anxiety	0.333	0.217	0.05	[0.11, 0.30]	4.33	**< 0.001**	**0.002**
Self‐consciousness	0.117	0.075	0.05	[−0.02, 0.17]	1.49	0.140	0.168
*Interpersonal Sensitivity*							
Age	−0.029	−0.012	0.02	[−0.04, 0.02]	−0.59	0.558	—
Sex at birth	0.142	0.146	0.11	[−0.07, 0.37]	1.33	0.186	—
Depression	0.160	0.182	0.11	[−0.04, 0.41]	1.65	0.101	—
Anxiety	0.127	0.152	0.10	[−0.05, 0.35]	1.52	0.132	—
Social anxiety	0.288	0.316	0.08	[0.15, 0.45]	3.95	**< 0.001**	**0.002**
Self‐consciousness	0.334	0.355	0.08	[0.20, 0.51]	4.44	**< 0.001**	**0.002**
*Interpersonal ambivalence*							
Age	−0.062	−0.008	0.01	[−0.04, 0.01]	−0.83	0.409	—
Sex at birth	−0.287	−0.161	0.09	[−0.33, 0.02]	−1.79	0.076	—
Depression	0.085	0.052	0.09	[−0.12, 0.23]	0.58	0.562	—
Anxiety	0.278	0.176	0.08	[0.02, 0.33]	2.20	**0.029**	—
Social anxiety	0.215	0.118	0.06	[−0.00, 0.24]	1.96	0.052	0.078
Self‐consciousness	−0.077	−0.041	0.06	[−0.17, 0.08]	−0.68	0.496	0.496
*Need for social approval*							
Age	−0.094	−0.039	0.02	[−0.07, 0.00]	−1.96	0.052	—
Sex at birth	0.101	0.108	0.11	[−0.12, 0.34]	0.98	0.329	—
Depression	0.289	0.370	0.12	[0.13, 0.58]	3.08	**0.002**	—
Anxiety	0.090	0.111	0.10	[−0.09, 0.32]	1.11	0.268	—
Social anxiety	0.310	0.351	0.08	[0.19, 0.50]	4.39	** <0.001**	**0.002**
Self‐consciousness	0.222	0.244	0.08	[0.09, 0.41]	3.05	**0.003**	**0.005**
*Lack of sociability*							
Age	−0.033	−0.008	0.01	[−0.04, 0.02]	−0.77	0.442	—
Sex at birth	0.017	0.019	0.10	[−0.17, 0.21]	0.19	0.851	—
Depression	0.303	0.369	0.10	[0.16, 0.54]	3.69	**< 0.001**	—
Anxiety	−0.083	−0.105	0.09	[−0.27, 0.07]	−1.17	0.246	—
Social anxiety	0.441	0.498	0.07	[0.34, 0.59]	7.12	**< 0.001**	**0.002**
Self‐consciousness	0.293	0.322	0.07	[0.18, 0.45]	4.60	**< 0.001**	**0.002**
*Quality of life*							
Age	0.021	0.001	0.00	[0.18, 0.45]	0.51	0.609	—
Sex at birth	0.163	0.019	0.01	[−0.00, 0.05]	1.88	0.062	—
Depression	−0.680	−0.086	0.01	[−0.14, −0.09]	−8.62	**< 0.001**	—
Anxiety	−0.092	−0.014	0.01	[−0.04, 0.01]	−1.35	0.180	—
Social anxiety	−0.105	−0.018	0.01	[−0.04, 0.00]	−1.77	0.079	0.105
Self‐consciousness	−0.070	−0.011	0.01	[−0.03, 0.01]	−1.14	0.258	0.281

*Note:* Each independent model is separated by lines with the outcome variable listed above the predictors and results. Covariates are listed first and the two primary predictors are at the bottom of each model. Standardized coefficients (β) are presented for interpretability; *SE*'s, CI's, *t*‐values, and *p*‐values correspond to unstandardized coefficients (*b*). Significant *p*‐values are bolded. *p*‐corrected values are presented for focal effects, and represent the *p*‐value after adjusting for false discovery rates (*q* = 0.05).

**Figure 1:**
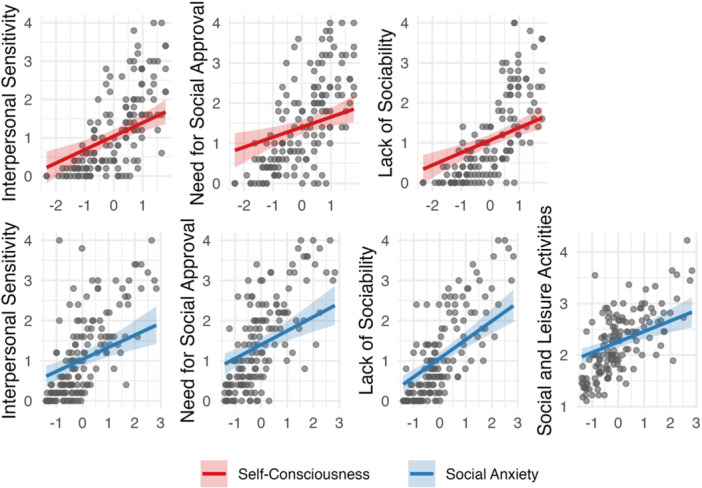
*All Significant Primary Predictors from Cross‐Sectional Analyses*. *Note:* Lines for each plot represent the effect of the primary predictor after adjusting for covariates (i.e., the effect reported in Table 1). These lines are overlayed on the raw datapoints, representing the simple bivariate relationship between the predictor and relevant outcome. Whereas Table 1 provides numerical model estimates, Figure 1 is intended to illustrate the form, magnitude, and variability of these relationships by plotting adjusted model lines over raw data points.

#### 2.1.1 Social Anxiety

We observed that SA significantly predicted unique variance in lack of sociability (*β* = 0.441, *p‐corrected* = 0.002, *η* ^2^ *p* = 0.26), interpersonal sensitivity (*β* = 0.288, *p‐corrected* = 0.002, *η* ^2^ *p* = 0.10), and need for social approval (*β* = 0.310, *p‐corrected* = 0.002, *η* ^2^ *p* = 0.12). SA was also associated with lower satisfaction with social and leisure behavior (*β* = 0.333, *p‐corrected* = 0.002, *η* ^2^ *p* = 0.12). In each case, higher scores on SA were associated with greater impairments at baseline, controlling for covariates including the depression and anxiety composites. SA was not a significant predictor of interpersonal ambivalence or overall quality of life.

#### 2.1.2 Self‐Consciousness

SC was incrementally associated with lack of sociability (*β* = 0.293, *p‐corrected* = 0.002, *η* ^2^ *p* = 0.13), interpersonal sensitivity (*β* = 0.334, *p‐corrected* = 0.002, *η* ^2^ *p* = 0.12), and need for social approval (*β* = 0.222, *p‐corrected* = 0.005, η^2^ *p* = 0.06). In each case, higher scores on SC were associated with greater impairments at baseline after adjusting for covariates. SC did not predict unique variance in social and leisure activity, interpersonal ambivalence, or overall quality of life.

All effects remained significant when restricting analyses to the internalizing sample.

### 2.2 Longitudinal Associations

**Figure 2:**
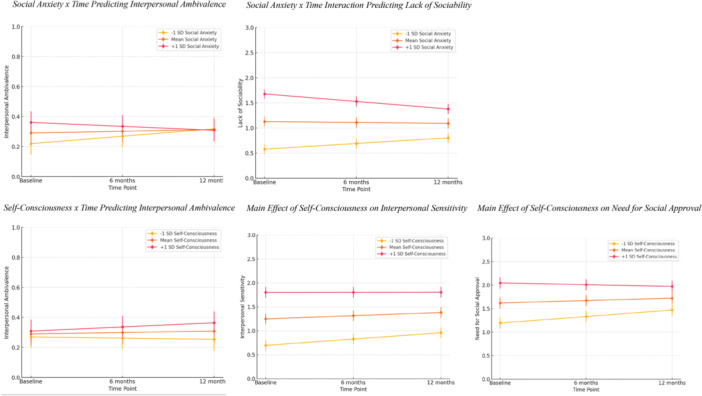
*All Significant Primary Predictors from Linear Mixed Model Analyses*. *Note:* All significant effects and time interactions for social anxiety and self‐consciousness in the longitudinal models. Time was centered at 12 months, so significant lower‐order effects reflect a significant effect of the predictor on the dependent variable at the 12‐month timepoint.

Full results for all linear mixed models can be viewed in Table 2, and a visualization of all significant relationships for primary predictors can be viewed in Figure 2. Across models, the fixed effect of time was generally small and often nonsignificant, suggesting modest average change in the functioning measures over follow‐up.

**Table 2:** Results from all linear mixed models in the full sample.

	β	*b*	*SE*	95% CI	*t*	*p*	*p corrected*
*Social and leisure activities*							
Time	−0.077	−0.060	0.03	[−0.11, −0.01]	−2.22	**0.028**	—
Sex at birth	−0.024	−0.029	0.06	[−0.15, 0.09]	−0.47	0.642	—
Age	−0.113	−0.021	0.01	[−0.04, −0.00]	−2.26	**0.025**	—
Depression	0.069	0.042	0.10	[−0.15, 0.23]	0.43	0.671	—
Anxiety	0.096	0.059	0.10	[−0.13, 0.25]	0.60	0.547	—
Social anxiety	0.134	0.074	0.07	[−0.05, 0.20]	1.14	0.257	0.400
Self‐consciousness	0.117	0.065	0.07	[−0.07, 0.20]	0.94	0.346	0.461
Time × Depression	−0.282	−0.116	0.06	[−0.23, −0.00]	−2.04	**0.043**	—
Time × Anxiety	0.086	0.036	0.06	[−0.07, 0.14]	0.65	0.520	—
Time × Social anxiety	−0.143	−0.052	0.04	[−0.13, 0.02]	−1.37	0.174	0.347
Time × Self‐consciousness	−0.034	−0.012	0.04	[−0.09, 0.07]	−0.31	0.761	0.773
*Interpersonal ambivalence*							
Time	0.026	0.011	0.03	[−0.04, 0.06]	0.44	0.659	—
Sex at birth	−0.076	−0.089	0.08	[−0.25, 0.07]	−1.09	0.279	—
Age	−0.087	−0.016	0.01	[−0.04, 0.01]	−1.30	0.195	—
Depression	−0.139	−0.082	0.11	[−0.30, 0.13]	−0.74	0.458	—
Anxiety	0.474	0.289	0.11	[0.08, 0.50]	2.68	**0.008**	—
Social anxiety	−0.182	−0.096	0.07	[−0.24, 0.05]	−1.31	0.191	0.347
Self‐consciousness	0.285	0.152	0.08	[0.00, 0.30]	1.97	0.050	0.128
Time × Depression	−0.180	−0.071	0.05	[−0.17, 0.03]	−1.35	0.181	—
Time × Anxiety	0.149	0.060	0.05	[−0.04, 0.16]	1.16	0.248	—
Time × Social anxiety	−0.261	−0.093	0.04	[−0.16, −0.02]	−2.65	**0.009**	**0.044**
Time × Self‐consciousness	0.270	0.096	0.04	[0.02, 0.17]	2.59	**0.011**	**0.044**
*Interpersonal sensitivity*							
Time	0.059	0.070	0.04	[−0.01, 0.15]	1.71	0.090	—
Sex at birth	0.093	0.208	0.11	[−0.01, 0.43]	1.87	0.064	—
Age	−0.022	−0.007	0.02	[−0.04, 0.02]	−0.45	0.652	—
Depression	−0.060	−0.066	0.16	[−0.39, 0.25]	−0.40	0.688	—
Anxiety	0.159	0.186	0.16	[−0.13, 0.50]	1.15	0.252	—
Social anxiety	0.060	0.061	0.11	[−0.15, 0.27]	0.57	0.570	0.633
Self‐consciousness	0.511	0.528	0.11	[0.30, 0.75]	4.61	**< 0.001**	**0.010**
Time × Depression	−0.136	−0.102	0.09	[−0.27, 0.07]	−1.18	0.239	—
Time × Anxiety	0.024	0.018	0.09	[−0.15, 0.19]	0.21	0.833	—
Time × Social anxiety	−0.166	−0.113	0.06	[−0.23, 0.00]	−1.96	0.051	0.128
Time × Self‐consciousness	0.100	0.069	0.06	[−0.05, 0.19]	1.12	0.265	0.400
*Need for social approval*							
Time	0.040	0.049	0.05	[−0.04, 0.14]	1.09	0.276	—
Sex at birth	0.090	0.216	0.12	[−0.02, 0.45]	1.83	0.069	—
Age	−0.074	−0.028	0.02	[−0.06, 0.01]	−1.59	0.115	—
Depression	0.187	0.230	0.18	[−0.12, 0.57]	1.31	0.192	—
Anxiety	0.096	0.120	0.17	[−0.22, 0.46]	0.68	0.495	—
Social anxiety	0.092	0.101	0.12	[−0.13, 0.33]	0.87	0.385	0.461
Self‐consciousness	0.276	0.304	0.12	[0.06, 0.55]	2.47	**0.014**	**0.047**
Time × Depression	−0.042	−0.033	0.10	[−0.22, 0.15]	−0.35	0.730	—
Time × Anxiety	−0.016	−0.014	0.09	[−0.20, 0.17]	−0.15	0.881	—
Time × Social anxiety	−0.157	−0.115	0.06	[−0.24, 0.01]	−1.81	0.072	0.160
Time × Self‐consciousness	0.027	0.020	0.07	[−0.11, 0.15]	0.29	0.773	0.773
*Lack of sociability*							
Time	−0.005	−0.015	0.04	[−0.09, 0.06]	−0.41	0.684	—
Sex at birth	0.046	0.100	0.09	[−0.08, 0.28]	1.08	0.282	—
Age	−0.014	−0.005	0.01	[−0.03, 0.02]	−0.33	0.740	—
Depression	0.206	0.228	0.14	[−0.05, 0.51]	1.61	0.110	—
Anxiety	−0.001	−0.001	0.14	[−0.28, 0.28]	−0.01	0.992	—
Social anxiety	0.102	0.101	0.09	[−0.08, 0.28]	1.08	0.280	0.400
Self‐consciousness	0.438	0.439	0.10	[0.24, 0.63]	4.40	**< 0.001**	**0.010**
Time × Depression	−0.060	−0.043	0.08	[−0.20, 0.11]	−0.55	0.584	—
Time × Anxiety	0.069	0.052	0.08	[−0.10, 0.20]	0.67	0.501	—
Time × Social anxiety	−0.256	−0.170	0.05	[−0.27, −0.07]	−3.22	**0.002**	**0.013**
Time × Self‐consciousness	0.073	0.048	0.06	[−0.06, 0.16]	0.86	0.392	0.461
*Quality of life*							
Time	0.075	0.015	0.01	[0.00, 0.03]	2.08	**0.039**	—
Sex at birth	0.044	0.014	0.01	[−0.01, 0.04]	1.00	0.321	—
Age	−0.003	−0.000	0.00	[−0.00, 0.00]	−0.06	0.953	—
Depression	−0.239	−0.039	0.02	[−0.09, 0.01]	−1.59	0.113	—
Anxiety	−0.117	−0.019	0.02	[−0.07, 0.03]	−0.79	0.432	—
Social anxiety	−0.062	−0.009	0.02	[−0.04, 0.02]	−0.58	0.564	0.570
Self‐consciousness	−0.076	−0.011	0.02	[−0.04, 0.02]	−0.66	0.512	0.569
Time × Depression	0.351	0.038	0.01	[0.01, 0.07]	2.54	**0.012**	—
Time × Anxiety	−0.053	−0.006	0.01	[−0.03, 0.02]	−0.39	0.697	—
Time × Social anxiety	0.012	0.001	0.01	[−0.02, 0.02]	0.10	0.921	0.768
Time × Self‐consciousness	0.032	0.003	0.01	[−0.02, 0.02]	0.29	0.769	0.672

*Note:* Each independent model is separated by lines with the outcome variable listed above the predictors and results. Standardized coefficients (β) are presented for interpretability; *SE*'s, CI's, *t*‐values, and *p*‐values correspond to unstandardized coefficients (*b*). Significant *p*‐values are bolded. *p*‐corrected values are presented for focal effects, and represent the *p*‐value after adjusting for false discovery rates (*q* = 0.05). Time was centered at 12 months to facilitate interpretation of the lower‐order effects.

#### 2.2.1 Social Anxiety

We did not observe any significant effects of SA in the longitudinal models, suggesting that SA at baseline did not prospectively predict any of the outcome variables 12 months later after adjusting for covariates. We observed significant time‐by‐SA interactions to predict both interpersonal ambivalence, *F*(1, 164) = 7.03, *p‐corrected* = 0.044, *η* ^2^ *p* = 0.041, and lack of sociability, *F*(1, 193) = 10.37, *p‐corrected* = 0.013, *η* ^2^ *p* = 0.051. In both cases, the pattern of the interaction suggested that the predictive power of SA on the relevant outcome diminished over time and was not present at the 12‐month follow‐up, which is consistent with a regression to the mean effect. These interaction patterns remained when restricting analyses to the internalizing sample.

#### 2.2.2 Self‐Consciousness

Our models yielded significant effects of SC on interpersonal sensitivity, *F*(1, 277) = 21.21, *p‐corrected* = 0.010, *η* ^2^ *p* = 0.071, need for social approval, *F*(1, 279) = 6.08, *p‐corrected* = 0.047, *η* ^2^ *p* = 0.021, and lack of sociability, *F*(1, 279) = 19.40, *p‐corrected* = 0.010, *η* ^2^ *p* = 0.065 at 12 Months. These effects suggested that SC assessed at baseline was still a significant prospective predictor of these variables 12 months later, controlling for covariates including depression and anxiety. We also observed a significant interaction between SC and time to predict interpersonal ambivalence, *F*(1, 167) = 6.69, *p‐corrected* = 0.044, *η* ^2^ *p* = 0.039. This suggested that, although there was no relationship at baseline, SC was a significant predictor of greater interpersonal problems (in the form of interpersonal ambivalence) 12 months later (see Figure 2).

These effects all remained in the internalizing‐only sample, with the exception of the lower order effect of SC on need for social approval.

## 3 Discussion

We explored the unique predictive power of social anxiety and self‐consciousness on social functioning and quality of life, both cross‐sectionally and prospectively over a 12‐month period. The two predictors were strongly correlated at baseline (i.e., *r* = 0.65); therefore, incremental effects of SC and SA in our regression models reflect variance unique to each construct, independent of the other and of general distress. In our cross‐sectional baseline models, both SA and SC predicted unique variance in interpersonal sensitivity, need for social approval, and lack of sociability, over and above internalizing symptom severity. Only SA was uniquely associated with social and leisure satisfaction at baseline. SA was not a significant predictor at 12 months for any outcome, while none of the SC effects disappeared over time, even after controlling for internalizing symptoms. These patterns, while preliminary, raise the possibility that SA and SC are non‐redundant forms of self‐focused attention that have different temporal associations with functional outcomes. SA may represent a more acute presentation of interpersonal difficulties that can normalize over time, whereas SC may be associated with deficits that are more stable.

How can we make sense of these findings in light of existing research? Social anxiety and SC are closely related forms of self‐focused attention, with SA characterized by fear of social judgment (Clark and Wells 1995; Rapee and Heimberg 1997) and SC by chronic feelings of shame or embarrassment (Panayiotou et al. 2017; Fournier et al. 2019). Cross‐sectional work has consistently linked both SA and broader self‐evaluative traits to poorer interpersonal functioning and quality of life (e.g., Buckner et al. 2012; Koyuncu et al. 2014; Sullivan et al. 2020), but most studies have examined these constructs separately, leaving it unclear whether impairment reflects shared variance or distinct liabilities. By modeling SA and SC simultaneously, our results provide tentative support for the idea that each construct is associated with distinct patterns of concurrent dysfunction. These findings complement work linking self‐evaluative traits to psychosocial outcomes (Fournier et al. 2019) while raising novel possibilities regarding their uniqueness and predictive potential.

The specific patterns of effects we observed in our longitudinal models (see Figure 2) might yield further insights into how these constructs are separable. The significant SA trajectories cleanly map onto regression to the mean patterns, where effects of SA on the outcome approach zero over time. This is less evident for the significant SC effects. While there is some regression to the mean (i.e., weaker effects of SC on the outcome over time) in the interpersonal sensitivity and need for social approval models, the effects are still present at 12 months. In the interpersonal ambivalence model, the effect of SC is actually strongest at 12 months. This is consistent with the idea that the aspects of SC that are unique and separable from SA and general internalizing may be associated with more stable deficits in interpersonal functioning. Unique interpersonal problems associated with SA, by contrast, might be more likely to resolve as general mental health and functioning improves.

These differences could relate to the specific type of self‐focused attention involved. Research distinguishes between private SC, the tendency to turn one's attention inward towards thoughts, feelings, and attitudes (e.g., somatic symptoms, worries, beliefs), and public SC, which involves outward attention on publicly observable aspects of the self (e.g., body language, facial expressions; Fenigstein et al. 1975; Clark and Wells 1995). Previous investigations have linked SA more strongly with public SC, whereas depression has shown stronger relationships with private SC (Mor and Winquist 2002). Measures of SA may therefore be capturing a specific form of self‐focused attention that is more dependent on external circumstances, and its associations with measures of functioning may be less enduring over time. Measures of SC, in contrast, could be capturing a more general trait‐like tendency towards self‐focused attention. These possibilities are hypothesis generating, and will need to be further explored in longitudinal designs that allow more precise examination of fluctuation in SA, SC, and functional outcomes over time.

Our findings, if replicated, could have important clinical implications. For example, therapeutic interventions targeting SA might produce more immediate improvements in social functioning, which could allow the clinician to shift the focus in long‐term care to relapse‐prevention strategies. This is supported by the large effect sizes of Cognitive Behavioral Therapy treatments for social anxiety, which are relatively enduring (Fogarty et al. 2019; Kindred et al. 2022). Viewing the consequences of SC as more trait‐like, on the other hand, might suggest that interventional techniques aimed at acceptance, adaptive coping, and broad emotion‐regulation strategies (e.g., Acceptance and Commitment Therapy, Dialectical Behavior Therapy), could be effective at altering the longer‐term trajectory of social functioning associated with SC. Effectively assessing and separating SC from SA may therefore allow practitioners to identify which patients are likely better suited for strategies aimed at creating shorter‐term changes in symptoms and which might need strategies aimed at altering more enduring features relevant to the individual's social functioning. If our findings are replicated, doing so may lead to better case conceptualization and more efficient treatment assignment.

At the same time, naturalistic stability in functional outcomes should not be equated with poor responsiveness to treatment. Social anxiety itself demonstrates moderate‐to‐high test–retest reliability (e.g., Baker et al. 2002), yet responds robustly to cognitive‐behavioral interventions with large and durable effects (Hoffman and Smits 2008; Mayo‐Wilson et al. 2014). Moreover, even relatively stable personality traits can show meaningful change following psychological interventions (Roberts et al. 2017). Because treatment studies rarely measure self‐consciousness directly, it remains unknown whether SC would demonstrate similar malleability, highlighting an important direction for future research. Importantly, the associations that we observed between SA, SC, and social functioning were present even after accounting for robust assessment of general depression and anxiety symptom severity. This suggests that these measures are providing clinical information that is not adequately captured during standard symptom assessment. Nevertheless, confident inferences about the clinical implications of these findings require focused testing in future research.

### 3.1 Limitations

Several limitations are important to consider. For instance, baseline correlations among several self‐report variables were high (including SA and SC), potentially stemming from common method variance or social desirability bias, which may render incremental SA/SC effects conservative and more prone to error. The framing of our SA measure (i.e., past week) and SC measure (i.e., in general) may have exaggerated state/trait effects between these two constructs, although the LSAS (the more state‐like measure) is known to be stable over time (e.g., *r* = 0.83 over 12 weeks; Baker et al. 2002). Additionally, some longitudinal associations may partly reflect continuity in SA and SC themselves rather than uniquely prospective effects on outcomes, and the observed time‐by‐predictor interactions could reflect natural symptom fluctuation, unmeasured mediators/moderators (e.g., life events or treatment seeking), or differential attrition. That said, the fact many of our longitudinal effects emerged for one predictor and not the other in otherwise identical models still highlights their apparently distinct predictive power in this sample. More generally, our relatively small sample size and homogeneity regarding racial and ethnic diversity limits statistical power and generalizability. Finally, our design prevents us from inferring causal directionality and tempers our inferences about temporal associations among SA, SC, and functional outcomes. Future studies should seek to replicate and extend this work by utilizing multiple data sources (clinical interviews, lab tasks, qualitative measures) and more extensive longitudinal/experimental designs in large and diverse samples.

## 4 Conclusions

To our knowledge, this is the first study to directly compare the impact of SA and SC across a sample of individuals with no internalizing symptoms to those with elevated depression and anxiety. While both constructs were uniquely related to worse interpersonal functioning, effects of SA appeared to dissipate over time whereas associations with SC were more stable. Findings from this work may ultimately advance our understanding of SA, SC, and internalizing disorders by providing a foundation for future research into how these two constructs relate to important aspects of social functioning. These distinctions could allow clinicians to more effectively separate features associated with shorter‐term interpersonal challenges from those associated with longer‐term problems, and therefore to select more targeted and appropriate treatments.

## Conflicts of Interest

Jay C. Fournier receives royalties from Guilford Press. The other authors declare no conflicts of interest.

## Supporting information

Supporting File

## Data Availability

The data that support the findings of this study are available on request from the corresponding author. The data are not publicly available due to privacy or ethical restrictions.
