# Supplementary material for: Distinct Longitudinal Contributions of Social Anxiety and Self‐Consciousness to Interpersonal Dysfunction
Source: J Clin Psychol. 2026 May 29;82(9):1434–43. doi: 10.1002/jclp.70155 (PMC13441325; doi:10.1002/jclp.70155)
Supplement: Supplementary file 1 [file JCLP-82-1434-s001.docx]

**Supplementary materials**

**Primary Predictor Variables**

***Liebowitz Social Anxiety Scale* (LSAS; Liebowitz, 1987).** The LSAS is a 24-item measure assessing fear and avoidance across social interaction and performance situations. Participants rated each situation separately for fear and avoidance on 4-point scales ranging from 0 (*none*) to 3 (*severe*), yielding Fear, Avoidance, and Total scores. Participants responded to situations such as “Talking to people in authority” and “Giving a report to a group.” Higher Total scores indicated greater social anxiety severity. The LSAS has demonstrated strong internal consistency, good test–retest reliability, and convergent validity with other measures of social anxiety severity (e.g., Baker et al., 2002).

***NEO Personality Inventory–Revised* (NEO PI-R; Costa & McCrae, 1992).** The NEO PI-R is a 240-item self-report measure assessing personality within the Five-Factor Model framework. Participants responded to statements such as “I rarely feel lonely or blue” (reverse-scored) on a 5-point scale from 1 (*strongly disagree*) to 5 (*strongly agree*). In the present study, we used the Self-Consciousness facet (N4) of the Neuroticism domain, which assesses tendencies toward embarrassment, shame, and concern about others’ evaluations. We computed N4 scores by summing item responses, with higher scores indicating greater trait self-consciousness.

**Primary Outcome Variables**

***Quality of Life Enjoyment and Satisfaction Questionnaire–Short Form* (Q-LES-Q-SF; Rapaport et al., 2005).** The Q-LES-Q-SF is a 16-item self-report measure assessing enjoyment and satisfaction across life domains during the past week. Participants responded to items such as overall life satisfaction during the past week on 5-point scales ranging from 1 (*very poor*) to 5 (*very good*). We computed total Q-LES-Q-SF scores by summing the first 14 items (range 14–70), with higher scores indicating greater quality of life. The Q-LES-Q-SF has demonstrated strong internal consistency and good test–retest reliability in clinical samples (Rapaport et al., 2005).

***Social Adjustment Scale–Self-Report* (SAS-SR; Weissman & Bothwell, 1976), Social and Leisure Subscale.** The SAS-SR assesses social functioning over the past two weeks across multiple role domains. In the present study, we used the Social and Leisure section, which assesses things like frequency of social contact and ability to confide in friends. Participants responded to items such as “How many friends have you seen or been in contact with… in the last 2 weeks?” on 5-category ordinal response options. We coded responses so that higher values reflected poorer social adjustment and computed Social/Leisure scores by summing item responses (range 9–45). When participants endorsed “Not applicable” options, we treated those responses as missing and prorated the subscale by multiplying the mean of completed items by 9.

***Inventory of Interpersonal Problems* (IIP; Horowitz et al., 2000).** The IIP assesses distress associated with interpersonal difficulties. Participants responded to items such as “It is hard for me to say ‘no’ to other people” and “I am too sensitive to rejection” by indicating how distressing each problem had been on a 5-point scale from 0 (*Not at all*) to 4 (*Extremely*). We computed IIP total scores by summing item responses, with higher scores indicating greater interpersonal problems. The IIP has demonstrated strong internal consistency and established construct validity (Horowitz et al., 2000).

**Covariates**

**Depression Composite (z-scored average)**

***Montgomery–Åsberg Depression Rating Scale* (MADRS; Montgomery & Åsberg, 1979).** The MADRS is a 10-item clinician-rated scale assessing core depressive symptoms. Clinicians rated items assessing domains such as apparent sadness and reported sadness on 7-point severity scales (0–6). We computed total MADRS scores by summing item ratings, with higher scores indicating greater depression severity. The MADRS has demonstrated strong reliability and sensitivity for detecting change in clinical trials.

***Hamilton Depression Rating Scale* (HAM-D; Hamilton, 1960).** The HAM-D is a clinician-administered measure assessing depressive symptoms over the past week. We scored the 17-item version (HAM-D-17). Clinicians rated items assessing domains such as depressed mood and guilt on symptom-specific ordinal scales, and we computed total HAM-D-17 scores by summing item ratings, with higher scores indicating greater depression severity. The HAM-D has extensive evidence supporting convergent validity, although critiques note limitations in content coverage and item-level reliability across samples.

***Quick Inventory of Depressive Symptoms–Self-Report* (QIDS-SR16; Rush et al., 2003).** The QIDS-SR16 assesses depressive symptom severity over the past week across DSM-consistent symptom domains. Participants responded to symptom items (e.g., sleep disturbance, concentration difficulties) using 4-point response options, and we computed total scores ranging from 0 to 27, with higher scores indicating greater depression severity. The QIDS-SR16 has demonstrated strong internal consistency and convergent validity with clinician-rated depression measures.

***Mood and Anxiety Symptom Questionnaire–Short Form* (MASQ-D30; Smith et al., 2000; Wardenaar et al., 2010).** The MASQ-D30 is a 30-item self-report measure assessing general distress, anhedonic depression, and anxious arousal over the past week. Participants responded to items by indicating how frequently they had felt emotions such as “Confused” and “Hopeless” over the past week, using a 5-point scales. We reverse-scored positively valenced anhedonic depression items (e.g., *Successful*, *Happy*, *Optimistic*, *Having fun*) and computed each subscale by summing the relevant items. When items were missing, we prorated each subscale by multiplying the mean of completed items by 10. We operationalized depression as the sum of anhedonic depression and general distress. The MASQ-D30 demonstrates good internal consistency and evidence of construct and convergent validity.

**Anxiety Composite (z-scored average)**

***Mood and Anxiety Symptom Questionnaire–Short Form* (MASQ-D30; Smith et al., 2000; Wardenaar et al., 2010).** We operationalized anxiety as the sum of anxious arousal and general distress. We computed each as summed subscale scores with prorating for missing items as described above. The MASQ-D30 anxiety-related scales show good internal consistency and discriminant validity relative to depressive symptom measures.

***Hamilton Anxiety Rating Scale* (HAM-A; Hamilton, 1959).** The HAM-A is a 14-item clinician-rated scale assessing somatic and psychological anxiety symptoms. Clinicians rated items assessing domains such as anxious mood and tension on 5-point severity scales (0–4), and we computed total scores by summing item ratings, with higher scores indicating greater anxiety severity. The HAM-A has longstanding evidence of clinical utility, and structured administration.

***PROMIS Emotional Distress–Anxiety Short Form v1.0–Anxiety 8a* (PROMIS-ED-A; Pilkonis et al., 2011).** The PROMIS Anxiety Short Form v1.0–Anxiety 8a is an 8-item self-report measure assessing fear, worry, and hyperarousal over the past 7 days. Participants responded to items such as “My worries overwhelmed me” on 5-point frequency scales from 1 (*Never*) to 5 (*Always*). We converted raw scores to PROMIS T-scores (*M* = 50, *SD* = 10), with higher scores indicating greater anxiety severity. PROMIS Anxiety short forms demonstrate strong reliability and measurement precision across a broad range of severity.

**Table S1.** Participant Demographics at Baseline

|  | Full Sample | | Internalizing Sample | |
| --- | --- | --- | --- | --- |
|  | *M* | *SD* | *M* | *SD* |
| Age | 22.43 | 3.20 | 22.36 | 3.14 |
|  | *n* | *%* | *n* | *%* |
| Sex |  |  |  |  |
| Male | 55 | 36.2% | 33 | 34.4% |
| Female | 97 | 63.8% | 63 | 65.6% |
| Ethnicity |  |  |  |  |
| Hispanic/Latino | 9 | 5.9% | 6 | 6.3% |
| Not Hispanic/Latino | 143 | 94.1% | 90 | 93.8% |
| Race |  |  |  |  |
| American Indian/Alaska Native | 2 | 1.3% | 2 | 2.1% |
| Asian | 29 | 19.1% | 19 | 19.8% |
| Black/African American | 20 | 13.2% | 15 | 15.6% |
| Native Hawaiian/Pacific Islander | 2 | 1.3% | 1 | 1.0% |
| White | 113 | 74.3% | 71 | 74.0% |
| Other/Not Known | 6 | 4.0% | 1 | 1.0% |

Note: Participants selected one or more racial identities

**Table S2.** Correlations at Baseline in the Full Sample

|  | Social Anxiety | Self-Consciousness | Depression | Anxiety |
| --- | --- | --- | --- | --- |
| Self-Consciousness | .651** | -- | -- | -- |
| Quality of Life | -.665** | -.684** | -.869** | -.724** |
| Interpersonal Sensitivity | .708** | .714** | .713** | .649** |
| Interpersonal Ambivalence | .381** | .284** | .386** | .407** |
| Need for Social Approval | .725** | .692** | .737** | .666** |
| Lack of Sociability | .791** | .748** | .746** | .613** |
| Depression | .687** | .721** | -- | .793** |
| Anxiety | .629** | .597** | .793** | -- |
| Social & Leisure | .688** | .626** | .722** | .599** |

Note: *p < .01, **p<.001

**Table S3.** Correlations at Baseline in the Internalizing Sample

|  | Social Anxiety | Self-Consciousness | Depression | Anxiety |
| --- | --- | --- | --- | --- |
| Self-Consciousness | .462** | -- | -- | -- |
| Quality of Life | -.468** | -.430** | -.781** | -.439** |
| Interpersonal Sensitivity | .568** | .577** | .538** | .395** |
| Interpersonal Ambivalence | .260* | .130 | .271* | .292* |
| Need for Social Approval | .582** | .518** | .536** | .390** |
| Lack of Sociability | .696** | .658** | .560** | .293* |
| Depression | .501** | .469** | -- | .446** |
| Anxiety | .371** | .189 | .446** | -- |
| Social & Leisure | .572** | .416** | .591** | .333** |

Note: *p < .01, **p<.001
